# Signatures of co-evolutionary host-pathogen interactions in the genome of the entomopathogenic nematode *Steinernema carpocapsae*

**DOI:** 10.1186/s12862-017-0935-x

**Published:** 2017-04-26

**Authors:** Mitzi Flores-Ponce, Miguel Vallebueno-Estrada, Eduardo González-Orozco, Hilda E. Ramos-Aboites, J. Noé García-Chávez, Nelson Simões, Rafael Montiel

**Affiliations:** 1 Laboratorio Nacional de Genómica para la Biodiversidad, Unidad de Genómica Avanzada, Centro de Investigación y de Estudios Avanzados del Instituto Politécnico Nacional, Km 9.6 Libramiento Norte Carretera Irapuato - León, Irapuato, Guanajuato Mexico; 2 CIRN/Departamento de Biologia, Universidade dos Açores, Rua Mãe de Deus, 13, 9500-321 Ponta Delgada, S. Miguel - Açores Portugal

**Keywords:** Arms race, Red Queen, Trench warfare, Positive selection, Balancing selection, Genomic scans, dN/dS, Tajima’s D

## Abstract

**Background:**

The entomopathogenic nematode *Steinernema carpocapsae* has been used worldwide as a biocontrol agent for insect pests, making it an interesting model for understanding parasite-host interactions. Two models propose that these interactions are co-evolutionary processes in such a way that equilibrium is never reached. In one model, known as “arms race”, new alleles in relevant genes are fixed in both host and pathogens by directional positive selection, producing recurrent and alternating selective sweeps. In the other model, known as“trench warfare”, persistent dynamic fluctuations in allele frequencies are sustained by balancing selection. There are some examples of genes evolving according to both models, however, it is not clear to what extent these interactions might alter genome-level evolutionary patterns and intraspecific diversity. Here we investigate some of these aspects by studying genomic variation in *S. carpocapsae* and other pathogenic and free-living nematodes from phylogenetic clades IV and V.

**Results:**

To look for signatures of an arms-race dynamic, we conducted massive scans to detect directional positive selection in interspecific data. In free-living nematodes, we detected a significantly higher proportion of genes with sites under positive selection than in parasitic nematodes. However, in these genes, we found more enriched Gene Ontology terms in parasites. To detect possible effects of dynamic polymorphisms interactions we looked for signatures of balancing selection in intraspecific genomic data. The observed distribution of Tajima’s D values in *S. carpocapsae* was more skewed to positive values and significantly different from the observed distribution in the free-living *Caenorhabditis briggsae*. Also, the proportion of significant positive values of Tajima’s D was elevated in genes that were differentially expressed after induction with insect tissues as compared to both non-differentially expressed genes and the global scan.

**Conclusions:**

Our study provides a first portrait of the effects that lifestyle might have in shaping the patterns of selection at the genomic level. An arms-race between hosts and pathogens seems to be affecting specific genetic functions but not necessarily increasing the number of positively selected genes. Trench warfare dynamics seem to be acting more generally in the genome, likely focusing on genes responding to the interaction, rather than targeting specific genetic functions.

**Electronic supplementary material:**

The online version of this article (doi:10.1186/s12862-017-0935-x) contains supplementary material, which is available to authorized users.

## Background

Nematodes are the most abundant type of animals on earth in terms of the number of individuals, being an ancient and diverse group [1]. Their diversity and abundance are the result of their extraordinary ability to adapt, small size, resistant cuticle, and simple body plan [2]. Nematodes have independently evolved parasitism several times in all major clades [3–5] and it has been proposed that understanding the genomic adaptations to parasitism in one clade could give insight into how parasitism has evolved across the phylum [5, 6]. Entomopathogenic nematodes (EPNs) represent an interesting group of parasitic nematodes, comprising the genera *Heterorhabditis* and *Steinernema*, which are lethal parasites of insects capable of infecting and killing a wide range of insects. Invertebrate parasitism evolved independently in these genera, which belong to different clades of the Nematoda phylogeny (clades V and IV, respectively [3]), thus representing an interesting case of convergent evolution [7]. *Steinernema carpocapsae* is one of the most well-known species of EPNs. It has been used worldwide as a biocontrol agent for insect pests and represents an interesting model to understanding parasite-host interactions [8, 9]. Sharing a symbiotic association with the entomopathogenic bacteria *Xenorhabdus nematophila,* it is also emerging as a model for mutualistic symbiosis [10]. It has also been suggested that an entomopathogenic Steinernematidae was the ancestor from which vertebrate-parasitic Strongyloidoids evolved [5], in the same way that Heterorhabditidae has been suggested to be the ancestor of vertebrate parasites of the Strongylomorphs group [5]. Therefore, nematodes from the *Steinernema* and *Heterorhabditis* genera can be useful as models to the mammal-parasitic nematodes. This evolutionary relationship can also help to understand host transitions in this and other clades of the nematode phylogeny.

Recent genomic studies in *Steinernema carpocapsae* have shown that specific evolutionary and functional signatures in its genome can be related to parasitism. These involve a set of expanded gene families likely involved in parasitism, orthologous genes shared with other parasitic nematodes not present in free-living species, ncRNA families reported to be enriched in parasites, and the expression of proteins putatively associated with parasitism and pathogenesis [11, 12]. These signatures are most likely the result of evolutionary interaction with the hosts and suggest an active role during the pathogenic process.

It is known that hosts and pathogens interact in such a way that an equilibrium is never reached [13], with hosts evolving under selective pressure to avoid pathogen infection and pathogens with the pressure to evade host defenses [14]. Thus, changes in gene frequencies as a result of selection acting on one species create selection for changes in gene frequencies in the other species [15]. Two of the co-evolutionary models proposed are selective sweeps and dynamic polymorphisms, both involving reciprocal changes in host and pathogens. Selective sweeps occur when new alleles appear, by mutation or migration, eventually becoming fixed within the population by directional positive selection. This model is known as the “arms race”. On the other hand, dynamic polymorphisms involve fluctuations in allele frequencies caused by selection and are inherently persistent, although fixation can occur as a result of genetic drift. This model is known as the “Red Queen” dynamics [15], or “trench warfare” [14], and genes in this model do evolve under balancing selection [14].

Well-known examples of a co-evolutionary “arms-race” dynamic are genes involved in immunity and defence [13, 14, 16], i.e., genes directly involved in the host-pathogen interaction. However, it is not clear to what extent these interactions might alter the evolutionary patterns at the genome level, or to what extent they might affect levels of intraspecific diversity. One expectation would be that depending on the number of genes participating in the interaction, the total number of genes with specific signatures of selection, either from positive or balancing selection, would increase in pathogens, as compared with genomes of non-pathogenic organisms. Another prediction would be that the number of genes with signals of selection will increase in genes participating in the interaction as compared with genes that do not participate in it. It might be difficult to find all of the genes involved in the host-pathogen interaction, but a first approximation can be obtained by inducing the pathogen with host tissues and identifying the differentially expressed genes (e.g., [17]).

Comparative population genomics is showing that linked selection plays an important role in both the overall genetic diversity of a species and the variation in diversity within the genome [18]. If the host-pathogen interaction is increasing the number of genes evolving under positive selection that in turn are responsible for an increased number of selective sweeps, then a reduction in diversity is expected in the pathogen when compared with non-pathogen genomes. On the other hand, if the effects of the interaction are more related to balancing selection, then the above-mentioned reduction in diversity will not be found. Of course, a portion of the genes might be under positive selection and a portion of them under balancing selection, cancelling each other’s effect on diversity. In any case, evolutionary genetic interactions might represent an additional determinant of genetic diversity.

Here we address some of these questions analysing the genome of the entomopathogenic nematode *Steinernema carpocapsae* (Nematoda clade IV). Through interspecific data, we look for ancient patterns of directional positive selection analysing nematodes of different lifestyles belonging to clade IV, and conducting a parallel analysis with nematodes belonging to clade V to give an independent assessment. At another level, using intraspecific data we look for more recent signatures of balancing or directional selection. To obtain clues regarding the impact of the parasitic lifestyle at genomic level, we compare our results with an analysis of a non-pathogenic nematode (*Caenorhabditis briggsae*). Also, we compare the patterns of selection in genes codifying differentially expressed proteins in *S. carpocapsae* after induction with insect tissues, with the patterns observed in genes for proteins that do not respond to this induction and with the more general patterns found in the massive scans.

## Results

### Positive selection in interspecific data

To assess differences in the impact of positive selection at a genomic level according to different lifestyles, we analysed two datasets, one from nematode clade IV and the other from clade V [3]. Each set included sequences from four nematodes. The dataset from clade IV, called C4N4, included the following species: the mycophagous *Bursaphelenchus xylophilus*, the free-living nematode *Panagrellus redivivus*, the entomopahtogenic *Steinernema carpocapsae*, and the vertebrate parasite *Strongyloides ratti*, and comprised 1552 orthologues. The dataset from clade V, C5N4, included the free-living nematodes *Caenorhabditis briggsae* and *Pristionchus pacificus*, the entomopathogenic *Heterorhabditis bacteriophora*, and the vertebrate parasite *Haemonchus contortus*, comprising 1510 orthologues. The number of genes analysed represents approximately 9.5% of the 16,333 genes estimated for *S. carpocapsae* [12] and 6.91% of the 21,850 genes estimated for *C. briggsae* (release WS244) [19].

**Fig. 1:**
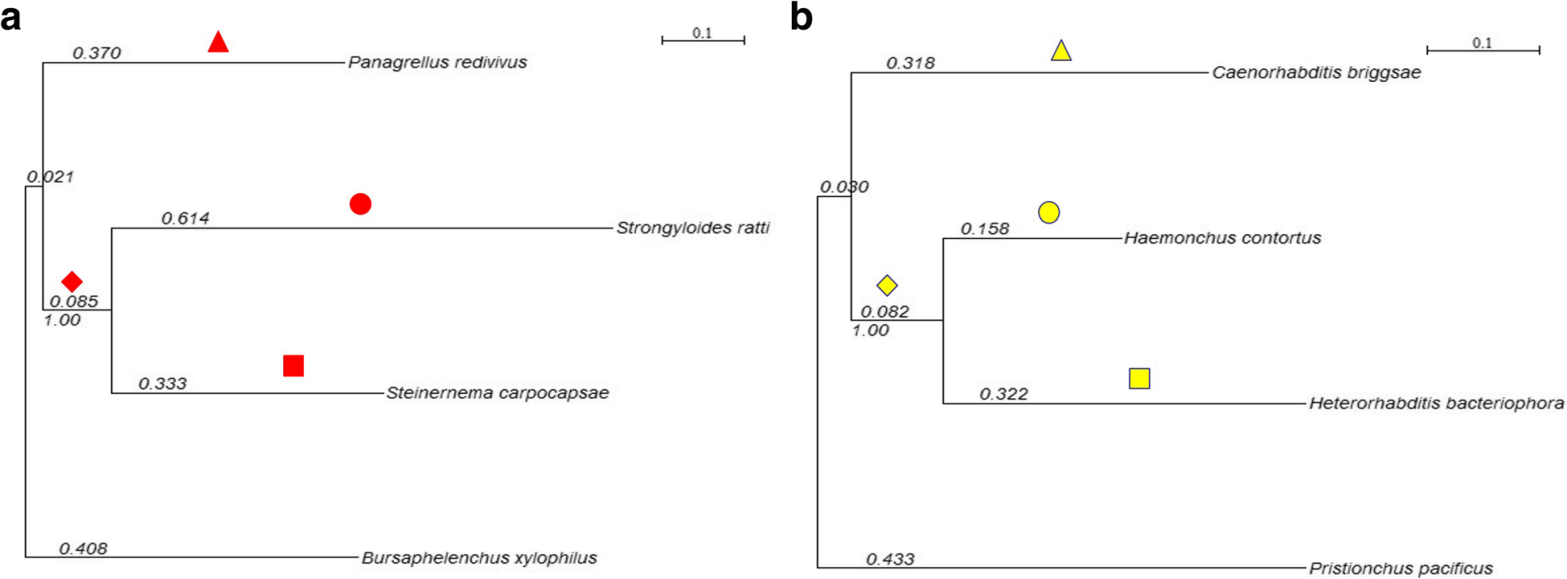
Phylogenetic reconstructions for each clade obtained from concatenated sequences for each dataset. **a** phylogenetic reconstruction from the 1552 orthologues in clade IV; **b** phylogenetic reconstruction from 1510 orthologues in clade V. Branches used for Likelihood Ratio Tests (LRTs) are marked according to the species lifestyles; ■, entomopathogenic; ●, vertebrate parasite; ♦ parasite branch; ▲, free-living. All tests were based on an unrooted phylogeny; the trees are rooted for display purposes only. Values correspond to branch lengths, and bootstrap values are shown under the branch leading to the animal parasites

Phylogenetic trees obtained from concatenated sequences for each dataset are shown in Fig. 1. Topologies are in agreement with previously published phylogenies [3, 12, 20].

We conducted massive scans of positive selection analysing ω (omega), the nonsynonymous/synonymous substitution rate ratio (dN/dS) [21, 22] (see Methods). We conducted several Likelihood-Ratio Tests (LRTs) for two of the existing codon models included in codeml, namely branch and branch-site models [23–26] (Table 1). For the branch model, we first compared the one-ratio model (model = 0), which assumes a single ω for all lineages, against the free-ratio model that assumes an independent ω for each lineage (model = 1). In the C4N4 set, 124 (7.99%) of the tested genes had a different ω value among branches (LRT, d.f. = 4, *p* < 0.05). In C5N4, the number of genes showing different ω values among branches was 113 (7.48%) (LRT, d.f. = 4, *p* < 0.05). Different values of ω among branches are indicative of episodic evolution, contrary to the neutral expectation [21]. In the following analyses, for each dataset we conducted four different LRTs, testing the branches of three different species and the branch leading to parasites. *Bursaphelenchus xylophilus* (clade IV) and *Pristionchus pacificus* (clade V) are basal in the phylogenies and their branches were not analysed in order to reduce the number of tests performed. In addition, *B. xylophilus* has a more complex lifestyle [27], complicating its comparison with other nematodes.

**Table 1:** Interspecific analyses of positive selection

Dataset name	C4N4 Clade IV				C5N4 Clade V			
Orthologues analysed (Global analysis)	1552 9.50%^a^				1510 6.91%^a^			
Genes with ω significantly different among branches (LRT, *p* < 0.05)	124 7.99%				113 7.48%			
Foreground branch (ω1)	Sc	Sr	(ScSr)	Pr	Hb	Hc	(HbHc)	Cb
Branch model								
Genes with ω1 > 1 and significantly different than ω0 (LRT, *p* < 0.05)	9 0.58%	11 0.71%	2 0.13%	5 0.32%	17 1.13%	11 0.73%	7 0.46%	17 1.13%
Genes with ω1 significantly greater than 1 (LRT, *p* < 0.05)	0	0	0	0	0	0	0	0
Branch-site model								
Genes with sites under positive selection (ω > 1) (LRT, *p* < 0.05)	74 4.77%	24 1.55%	21 1.3% 5	91 5.86%	61 4.04%	55 3.64%	20 1.32%	87 5.76%
Average proportion of sites under positive selection per gene (standard deviation)	4.94%	6.31%	3.58%	5.16%	9.88%	5.81%	8.74%	7.90%
	(0.053)	(0.117)	(0.045)	(0.054)	(0.140)	(0.068)	(0.155)	(0.092)

Percentages are from the total of genes tested for each set, unless stated

^a^Percentage of genes in relation to the total genes estimated for *S. carpocapsa*e [12] in clade IV and *C. briggsae* [19] in clade V. Sc, *Steinernema carpocapsae*; Sr, *Strongyloides ratti*; Pr, *Panagrellus redivivus;* Hb, *Heterorhabditis bacteriophora;* Hc, *Haemonchus contortus*; Cb, *Caenorhabditis briggsae*

We next tested the one-ratio model (model = 0) against a several ω ratios model that estimates one ω as a free parameter for each of the specified branches and one background ω for the remaining branches (model = 2). In total, we found 79 genes in which the selected branch had an estimated ω > 1 and a significant different value to the background ω (LRT, d.f. = 1, *p* < 0.05). From these, 27 (1.73%) were from C4N4 and 52 (3.44%) from C5N4. In the C4N4 set, the *S. carpocapsae* branch had 9 genes (0.58%) with these characteristics. For the remaining tested branches, the percentage of genes ranged from 0.13 to 0.71% in C4N4 and 0.46–1.13% in C5N4 (Table 1). To test if ω was significantly greater than 1 in these cases, we contrasted a two-ratio model with ω fixed to 1 for the specified branch and a freely estimated ω for the remaining branches (model = 2 – fix ω), against a two-ratio model as described above (model = 2). None of the genes tested had a ω significantly different from 1 (LRT, d.f. = 1, *p* > 0.05).

The branch models are conservative because positive selection often acts on one, or a few amino acids, and averaging ω over sites results in a lack of power [24, 25]. Therefore, we also used branch-site models, devised to detect positive selection affecting just a few sites along particular lineages [24, 25]. We compared the branch-site model A (specified using model = 2 and NSsites = 2), against the same model with the difference that ω_2_ was fixed to 1 (model A1). Using a LRT we assessed if ω was significantly different from ω_2_ (i.e., different from 1). For the C4N4 set, we found 74 (4.77%) genes with sites evolving under positive selection in the *S. carpocapsae* branch (LRT, d.f. = 1, *p* < 0.05), 24 (1.55%) in the *Strongyloides ratti* branch (LRT, d.f. = 1, *p* < 0.05), and 91 (5.86%) in the *Panagrellus redivivus* branch (Table 1). In the branch leading to parasites (*S. carpocapsae* and *Strongyloides ratti*), we found 21 (1.35%) genes with sites under positive selection (LRT, d.f. = 1, *p* < 0.05). In the C5N4 set we found 61 (4.04%) genes with sites under positive selection in the *Heterorhabditis bacteriophora* branch (LRT, d.f. = 1, *p* < 0.05), 55 (3.64%) in the *Haemonchus contortus* branch (LRT, d.f. = 1, *p* < 0.05), and 87 (5.76%) in the *Caenorhabditis briggsae* branch (LRT, d.f. = 1, *p* < 0.05) (Table 1). In the branch leading to parasites (*Heterorhabditis bacteriophora* and *Haemonchus contortus*), we found 20 (1.32%) genes with sites evolving under positive selection (LRT, d.f. = 1, *p* < 0.05). As can be seen, vertebrate parasites showed some variation in the proportion of these genes between clades (1.55% versus 3.64%) but not entomopathogenic (4.77% versus 4.04%) or free-living (5.86% versus 5.76%) nematodes (Table 1). However, when grouping species by the two main lifestyles, free-living and parasites, we found that parasitic nematodes showed lower proportions of genes with sites under positive selection (214/6124 = 3.49%) than free-living nematodes (178/3062 = 5.81%) (here the numbers in the denominators represent the total number of genes analysed for each lifestyle across clades). The difference between these proportion was highly significant (*χ* ^2^ = 26.87, d.f. = 1, *p* < 0.0000003).

**Fig. 2:**
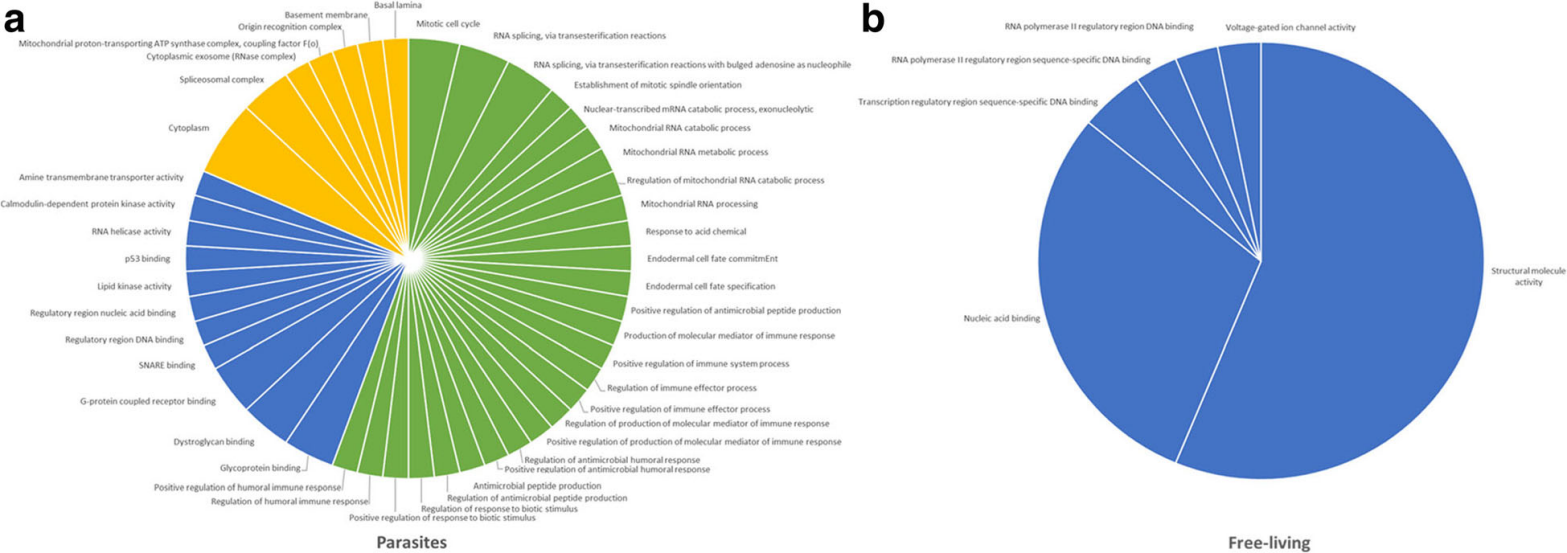
Over-represented unique GO terms in genes with sites under positive selection. **a** clade IV, **b** clade V. Biological process in *green*, molecular function in *blue*, and cellular component in *yellow*. Enrichment analyses were performed with Blast2GO with a Fisher’s exact test [65]

Functional annotation of genes with sites under positive selection is shown in Additional files 1 and 2. In this analysis each gene is associated to one or more Gene Ontology (GO) terms. We found 26 significantly over-represented (enriched) unique GO terms among the 210 protein-coding genes with signatures of positive selection (Additional file 3) in comparison to the remaining genes (Fisher’s exact test; *p* < 0.05). From these, 19 were enriched only in *S. carpocapsae*, one in *Strongyloides ratti*, five in *P. redivivus*, and only one was shared among the three species. In the 223 total protein-coding genes of clade V, we found 28 significantly enriched unique GO terms (Additional file 4), six of which were private to *Heterorhabditis bacteriophora*, 18 were found only in *Haemonchus contortus*, and four were shared in both nematodes. No enriched term was found among the *C. briggsae* genes with signs of positive selection. Only one GO term was found to be enriched in both clades IV and V (GO:0005198, structural molecule activity); therefore, there were 53 unique enriched terms considering both clades. In general, in parasitic nematodes the total number of enriched terms (47) was strikingly high in relation to free-living nematodes (6), even considering that the number of genes with signatures of positive selection was lower in parasitic nematodes (Additional files 3 and 4). Moreover, in parasitic nematodes, categories in cellular components, molecular function, and biological processes were represented, while in free-living nematodes, only categories related to molecular function were observed (Fig. 2). Most of the enriched terms in *S. carpocapsae* are related to the immune response or antimicrobial peptide production (Additional file 5). One example is the *S. carpocapsae* gene snf-12 (g2184.t1), encoding for the transmembrane protein SNF-12 (Uniprot O45813), a sodium-dependent neurotransmitter symporter, member of the solute carrier 6 family (SLC6), reported to be essential for two immune signalling pathways (neuroendocrine TGF-b pathway and a p38 MAPK pathway) in *C. elegans* [28]. The enrichment analyses showed only one term related to mitochondrial function found in *S. carpocapsae*; however, in the parasite *H. contortus* an important number of terms related to mitochondria were observed (Fig. 2, Additional files 3 and 4).

### Signatures of selection in intraspecific data

To conduct this analysis at genome level, we sequenced the genomes of four additional *S. carpocapsae* strains. To further assess the impact that different lifestyles could have in the selection patterns across the genome, we did the same analysis on previously published data from five strains of *C. briggsae* isolated from around the world [29]. A description of the strains used is provided in the Methods section.

We conducted tests of neutrality with the Tajima’s D statistic in sliding windows of 1000 bp across the genome, including both genic and intergenic regions. Significant positive values of Tajima’s D are indicative of balancing selection or could be caused by demographic processes that increase genetic variation [30, 31], whereas significant negative values are indicative of directional selection or could be caused by demographic processes reducing genetic variation, such as a recent bottleneck [30–32]. Directional selection can be due to either positive or negative selection [13]. To account for demographic effects, we used the observed distribution of the data to correct the Tajima’s D confidence limits to identify windows with significant values (see Methods).

**Fig. 3:**
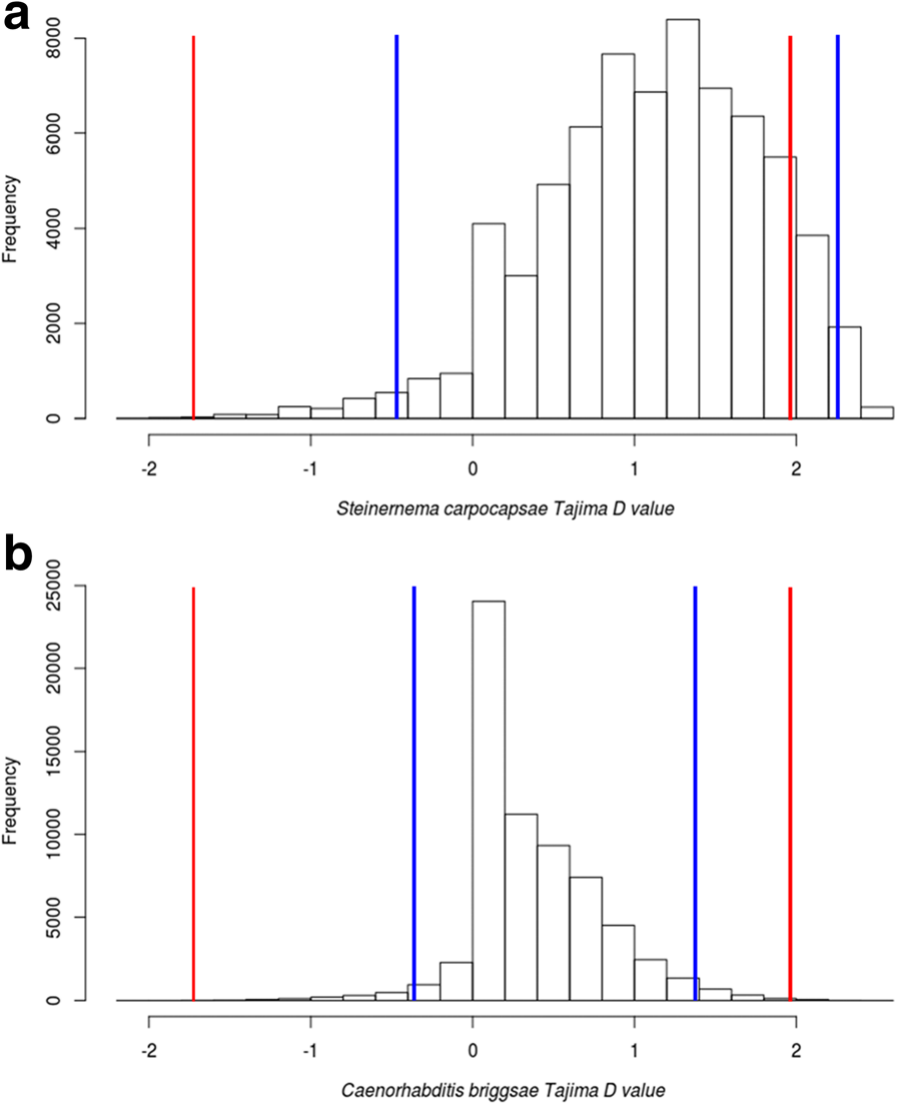
Distribution of Tajima’s D values estimated in sliding windows of 1,000 bp across the genomes. **a** *Steinernema carpocapsae;* **b** *Caenorhabditis briggsae*. D values were non-continuous (i.e., were distributed in discrete intervals). *Red lines* correspond to the maximum and minimum confidence limits according to [30]. *Blue lines* correspond to the maximum and minimum corrected confidence limits from the observed distribution (upper and lower 2%)

From the *S. carpocapsae* assembled genome, we analysed 84,767 windows, from which 69,694 had enough coverage to conduct the analysis (Table 2). From these, 400 windows (0.47%) were invariable (in these windows the estimation of Tajima’s D is not possible). The remaining 69,294 windows, which included 14,994 protein-coding genes (representing 91.84% of the protein-coding genome), were used to build the observed distribution of Tajima’s D values. These values ranged from −2.105 to 2.592. The corrected confidence limits for *S. carpocapsae* were −0.468 for negative and 2.264 for positive values, which differed from the uncorrected limits obtained with the Tajima’s beta distribution (−1.733 and 1.975) (Fig. 3). Using the corrected limits, we found 2,759 windows with significant D values, which included 530 genes (3.53% of the genes analysed). From these, 1,375 windows presented significant positive values, comprising 228 protein-coding genes (1.52% of the genes analysed) (Additional file 6). The *S. carpocapsae* gene (g6196t.1) with the most positive D value is orthologous to the *C. elegans* nuo-6 gene, coding for a subunit of the mitochondrial NADH dehydrogenase (ubiquinone) complex (complex I) (Uniprot Q23098). Meanwhile, 1,384 windows presented significant negative values, comprising 302 protein-coding genes (2.01%) (Additional file 6). The gene with the most negative D value corresponded to a *S. carpocapsae* gene (g10336.t1) that is orthologous to the *C. elegans* gen sut-1, coding for a protein involved in embryo development (Uniprot H2KYJ0). Additional file 6 contains brief descriptions of all the genes with significant D values. The number of windows with significant values according to the theoretical distribution are shown in Table 2 for comparison.

**Table 2:** Intraspecific analyses of selection

Species	*S. carpocapsae*	*C. briggsae*
Number of windows	84,767	108,421
Windows with coverage > 90%	69,694	65,933
Invariable windows	400	21
Windows with Tajima’s D values	69,294	65,912
Genes covered (>50%) in windows with D values (Global number of genes analysed)	14,994	15,473
Tajima’s D range of values	−2.105 — 2.592	−2.057 — 2.452
Tajima’s D average	1.097	0.355
Confidence limits according to theoretical distribution [30]	−1.733 — 1.975	−1.733 — 1.975
Theoretical significant windows	6522 (6500 positive, 22 negative)	80 (74 positive, 6 negative)
Confidence limits obtained from the observed distribution	−0.468 — 2.264	−0.355 — 1.369
Significant windows according to the observed distribution^a^	2,759 (1,375 positive, 1,384 negative)	2,624 (1,313 positive, 1,311 negative)
Genes in windows with significant D values^a^	530 (3.53%)	487 (3.15%)
- Significant positive	228 (1.52%)	247 (1.6%)
- Significant negative	302 (2.01%)	240 (1.55%)
π values range	0.00035 — 0.075	0.00035 — 0.045
Average π value	0.006700	0.007763
ϴ values range	0.35349 – 61.5065	0.3535 – 49.1345
Average ϴ value	5.369212	7.166656

^a^ Windows falling in the upper (positive) and lower (negative) 2% of the observed distribution

For *C. briggsae* we analysed 108,421 windows, from which 65,933 had enough coverage to conduct the analysis (Table 2). From these, 21 windows (0.02%) were invariable. The remaining 65,912 windows, which included 15,473 protein-coding genes (representing 66.52% of the protein-coding genome) were used to build the observed distribution of Tajima’s D values. D values ranged from −2.057 to 2.452 for this distribution. In *C. briggsae*, the confidence limits from the observed data distribution (−0.355 and 1.369) also differed from the confidence limits for the theoretical beta distribution (−1.733 and 1.975) (Fig. 3). We found 1,313 windows with significant positive values, comprising 247 protein-coding genes (1.6% of the genes analysed). The significant negative values included 1,311 windows comprising 240 protein coding genes (1.55%) (Table 2). The total number of genes with significant D values was 487 (3.15% of the genes analysed). The comparison with results using the theoretical distribution are shown in Table 2.

The mean of Tajima’s D values in *S. carpocapsae* was bigger (1.097) and significantly different from the average in *C. briggsae* (0.355) (W = 3759800000, *p* < 2.2 × 10^−16^). The difference in the average values might be caused by natural selection or by demographic processes. However, this difference was not due to a simple displacement of the distribution of one species in relation to the other, but to a clear change in the shape of the distribution. In fact, the distribution of Tajima’s D values were significantly different between these species (K-S test, D = 0.54736 *p* < 2.2 × 10^−16^), with the distribution of *S. carpocapsae* more skewed to positive values (Fig. 3).

Other statistically significant differences between *S. carpocapsae* and *C. briggsae* included the proportion of invariable windows (*χ* ^2^ = 321.74, d.f. = 1, *p* < 6.1 × 10^−72^), and the theta average (t = −82.681, d.f. = 134130, *p* < 2.2 × 10^−16^) (Table 2).

Functional annotations of genes in windows with significant Tajima’s D values are shown in Additional files 7 and 8. In *S. carpocapsae*, there were no enriched GO terms in the genes with significant values, either positives or negatives.

### Selection in differentially expressed proteins

To assess how selection is affecting genes related to the host-pathogen interaction, we analysed patterns of selection in genes for differentially expressed proteins. In a previous study, nematodes in the Infective Juvenile (IJ) stage were induced with host insect (*Galleria mellonella*) tissues to detect differentially expressed proteins [12]. In that study, we conducted a qualitative analysis with which we identified 1,154 differentially expressed proteins. In the present study, using the same data, we conducted a label-free quantitative analysis (see Methods). We identified 147 significantly differentially expressed proteins in relation to the control. Considering both qualitative and quantitative analyses, there were 1301 differentially expressed proteins (Additional file 9). In addition, we detected 2,237 proteins that were not differentially expressed between samples and the control (Additional file 10).

We then compared the results of the differential expression analysis with the results obtained from the analyses of selection with both interspecific and intraspecific data to assess if the patterns of selection in the differentially expressed genes were different in relation to the patterns in non-differentially expressed genes, and in relation to the global scans. We obtained data for 590 genes of the identified proteins in our interspecific scan for positive selection using the branch-site tests, including 143 differentially expressed and 447 non-differentially expressed genes. From these, we found 7.69% (11/143) differentially expressed genes with sites under positive selection, a higher percentage than the 6.71% (30/447) found in genes that were not differentially expressed (Table 3) (Additional file 11), although the difference was not statistically significant (Fisher’s exact test, *p* = 0.706381). Also, differentially expressed genes had a higher proportion of genes under selection (7.69%) in relation to the 4.77% in the global analysis (74/1552) (Table 1), but again the difference was not statistically significant (Fisher’s exact test, *p* = 0.15624).

**Table 3:** Patterns of selection in the genome of *Steinernema carpocapsae* and in identified expressed proteins

Interspecific analysis	*N*		Positive selection^a^	
Differentially expressed	143		11 (7.69%)	
Non-differentially expressed	447		30 (6.71%)	
Intraspecific analysis	N	Significant D^b^	Significant positive D^c^	Significant negative D^c^
Differentially expressed	1009	42 (4.16%)	23 (2.28%)	19 (1.88%)
Non-differentially expressed	2084	79 (3.80%)	29 (1.40%)	50 (2.40%)

*N* number of genes analysed

^a^Branch-sites test, LRT, *p* < 0.05

^b^Tajima’s D, 96% confidence level obtained from the real distribution

^c^Tajima’s D, 98% confidence level obtained from the real distribution

In the intraspecific analysis, we obtained data for 3093 genes of the identified proteins (1009 differential and 2084 non-differential). Among the differentially expressed proteins, 4.16% (42/1009) presented significant Tajima’s D values in their coding genes. From these, 2.28% (23/1009) presented positive D values, and 1.88% (19/1009) presented negative D values. In non-differentially expressed proteins, we found 3.8% (79/2084) significant D values, 1.4% (29/2084) with positive values, and 2.4% (50/2084) with negative values (Table 3) (Additional file 11). Differentially expressed genes showed a slightly higher proportion of genes with significant D values (4.16%) in relation to the 3.53% (530/14994) in the global scan (Table 2) and the non-differentially expressed genes (3.8%), although the differences were not significant (Fisher’s exact test, *p* = 0.292928 and *p* = 0.621698, respectively). Differentially expressed genes with significant positive D values (2.28%) showed also an increased proportion in relation to the 1.52% (228/14994) in the global scan (Table 2) and the non-differentially expressed genes (1.4%). Although the *p*-values were marginal (Fisher’s exact test, *p* = 0.066236 and *p* = 0.075006, respectively), these differences were not significant.

## Discussion

This study is a first assessment of the impact that parasitic lifestyle might have on nematode genomes. We characterized selection patterns at the genome level in parasitic and free-living nematodes from two clades of the Nematoda phylogeny to assess consistency. In addition, we assessed whether genes responding to the interaction with host tissues showed specific patterns of selection, using the entomopathogenic nematode *S. carpocapsae* as a model.

In the interspecific analysis, the observed proportions of genes with positively selected sites found in parasites as a whole and in free-living nematodes were similar in both clades (Table 1), despite differences in vertebrate parasites. Although we detected a significantly higher proportion of genes with sites under positive selection in free-living nematodes, functional enrichment tests showed a higher number of enriched GO terms and a different enrichment profile in parasites (Additional file 3: Figure S2), indicating that the difference is rather qualitative. This gives further support to previous observations indicating that an arms-race interaction between hosts and pathogens affects specific genetic functions, but does not necessarily increase the global number of positively selected genes [12]. In parasites, most of the enriched functions involved immune response, production of antimicrobial compounds, and mitochondrial processes (Fig. 2, Additional files 3 and 4). It is known that symbiotic entomopathogenic bacteria produce antimicrobial peptides during infection to eliminate other potential microbial pathogens from the host [33]. Nematodes could be contributing to this end with their own antimicrobial arsenal. In addition, immune response might be needed to fight against attacks from these same microbes or even to defend themselves from their exponentially growing symbiont, also during the infection process. In *C. elegans*, snf-12, homologous to one of the genes we identified in *S. carpocapsae*, has a role in defence against bacterial and fungi pathogens [28]). As there are no known symbionts in *C. elegans*, both possibilities, defence against other pathogens and against the symbiont remain open in EPNs. Enrichment in mitochondrial processes is interesting because there are few examples in which genes related to mitochondrial processes might be linked to parasitism [12]. Genes relevant to the mitochondrial function were also identified here in the intraspecific analysis. One of them, nuo-6, has been used for phylogenetic analysis in *Taenia* spp. and other species [34], although so far we did not find reports indicating any role in pathogenesis. The other gene, sut-1, is involved in development [35], as is ATAD-3, a protein previously identified as potentially relevant in the pathogenic process [12]. This could suggests that developmental processes are relevant in pathogenesis, a reasonable expectation considering that during the initial steps of infection nematodes need to recover from the resistance larval stage (IJ) to the more active parasitic stage [36]. The evaluation of different alleles or mutations in these genes to observe whether there are differential phenotypes in the pathogenic process could be a promising future area of research.

Interestingly, in both clades, the branches leading to parasitic nematodes (*S. carpocapsae* and *Strongyloides ratti* in clade IV and *Heterorhabditis bacteriophora* and *Haemonchus contortus* in clade V) had lower proportions of genes with sites evolving under positive selection than the branches of each of the parasitic species. This is difficult to interpret, however the increase in this proportion in each of the species might be related to host-shifting and specialization, mediated by coevolutionary dynamics.

In our interspecific analyses we scanned relatively low numbers of genes (between 7 and 9% of the protein-coding genomes) because nematode species are in general highly divergent [37], making it difficult to find a high number of one-to-one orthologues. Also, the genes analysed can be considered to be among the most conserved, and it can be counterintuitive to look for positive selection in these genes. However, these genes are especially interesting because they show variation at the sequence level while preserving their function, making them more relevant to assessing whether the observed differences between the species were fixed by positive selection or by random drift [38, 39]. In our case, all but one of the analysed orthologues showed variation among the species at the amino acid level but only a small proportion of them (1.3 – 5.76%) showed signs of positive selection. This leaves open the possibility that most of the observed amino acid variation is neutral, or is due to linked selection, possibilities that need to be investigated further. In other studies, the proportion of genes under positive selection varied between 2.4% in mammals [16] to 43% in ants [40], also indicating that substantial amounts of variation are not explained by positive selection.

To conduct a more comprehensive analysis of the effect that lifestyle might have at genomic level, including coding and intergenic regions, an intraspecific analysis is appropriate. With this analysis, more recent or ongoing selection events can be detected [13]. Demographic processes might also produce signals that can be confounded with signatures of selection, reflected in positive D values (for processes increasing diversity) or in negative D values (for processes decreasing diversity) [13, 30, 32]. However, demographic events are expected to affect all genes in a genome, whereas selection pressures act in specific loci of the genome [30, 32, 41]. Thus, a method to discern demographic from selective effects is to conduct a genome-wide analysis. Better results can be achieved if specific population parameters can be modelled for the specific populations under analysis [42], although this requires a good characterization of the variation in the populations that is currently lacking for nematode species. Our strategy allowed us to obtain an observed distribution from which we estimated corrected confidence limits to detect Tajima’s D values that were significantly different from both the expected values under neutrality and the values linked to demographic processes, which were assumed to be reflected in the average of D values across all the analysed windows. The comparison of these confidence limits with those obtained from the theoretical distribution proposed by Tajima [30] indicated that our approach avoided a bias in accepting or rejecting candidate regions. The Tajima confidence limits are asymmetrical in relation to the real data distribution (Fig. 3). Thus, the estimation of confidence limits from the real distribution of D values improves the power of the test and avoids bias towards positive or negative values. A similar result was obtained by Schmidt and Pool [43] using simulations. One problem of our approach is that when performing comparisons between species, the percentage of significant positive and negative values will always be similar, because the idea is to accept a predefined marginal percentage of values from each side of the distribution (in our case, 2%). This nullifies any possible conclusion regarding the impact of lifestyle in the proportion of genes with signatures of selection. Nevertheless, the comparison of the distribution itself can gives us some clues regarding the impact of lifestyle in the patterns of selection at the genomic level. The shape of the distribution of *S. carpocapsae* is different and more skewed to positive values of Tajima’s D in relation to the distribution of *C. briggsae* (Fig. 3). This might be indicative of pervasive balancing selection acting in the genome of the entomopathogenic nematode, due to Red Queen or trench warfare co-evolutionary host-pathogen dynamics. An alternative explanation is that demographic processes are not only affecting the average D value, but also changing the shape of the distribution, a more complex scenario that needs to be further investigated; perhaps by modelling different demographic scenarios to assess specific effects in Tajima’s D distribution. Another problem in our comparison is that there is more genetic distance among the *C. briggsae* strains than among *S. carpocapsae* strains included in the analyses (Additional file 12). This is reflected in the significantly higher number of invariable windows detected in *S. carpocasae* in relation to *C. briggsae*. However, positive values of Tajima’s D correlate with higher levels of nucleotide diversity in relation to segregating sites [30]. Therefore, the fact that *S. carpocapsae* shows more windows with positive D values than *C. briggsae,* when there is less genetic distance among their strains, reinforces the idea that balancing selection is acting with more strength in *S. carpocapsae* due to its pathogenic lifestyle. Under balancing selection, more alleles will be maintained in each segregating site. *S. carpocapsae* has a significant lower theta (θ) average than *C. briggsae* (Table 2), an estimator that is proportional to the number of segregating sites [30]. To maintain different alleles in a significant number of these sites, a higher strength of selection is required.

To assess if genes participating in the interaction with the host show different patterns of selection, we compared the proportion of genes with signatures of selection in differentially expressed genes against the proportion found in the global analyses and with the proportion of non-differentially expressed genes (Table 3). In the interspecific analysis, the proportion of genes with positive selected sites was higher in differentially expressed genes (7.69%) in relation to both, the global analysis (4.77%) and the non-differentially expressed genes (6.71%), although the differences were not statistically significant. Interspecific analyses are suitable for detecting past selection [13] in a time frame commensurate with the divergence time of the species analysed. In our analysis, we detected ancient selection and it is possible that the genes that were targeted at that time are different than the genes that are currently relevant in the interaction. In the intraspecific analyses, we observed a slightly higher proportion of genes with significant D values among the differentially expressed genes (4.16%) in relation to both the global scan (3.53%) and to the non-differentially expressed genes (3.80%) that were not significant. Interestingly, the differences were more evident in the proportion of genes with significant positive D values. There were 2.28% of these genes among the differentially expressed genes, 1.52% in the global scan, and 1.40% in the non-differentially expressed genes. It is also interesting that the tendency is reverted in the proportion of genes with significant negative D values, which is lower in differentially expressed genes (1.88%) than in the global scan (2.01%) or in the non-differentially expressed genes (2.4%).

The lack of significance in the observed differences precludes further conclusions. In the case of the interspecific analysis, a clearer picture might emerge by analysing more closely related species. Also, both analysis were based on protein-coding expressed genes detected under specific conditions and methods. Testing for different conditions, aiming to capture different stages during the pathogenic process or specific groups of genes, might increase the power of the tests and help to tease apart the relevance of specific types of selection along the process.

## Conclusions

Our study provides a first portrait of the effects that lifestyle might have in shaping the patterns of selection at the genomic level. Our massive scan for positive selection indicates that in pathogenic nematodes, positive selection is targeting specific genetic functions, possibly due to an arms-race host-pathogen interaction. Our intraspecific genomic analysis indicates that balancing selection could be acting with more strength in the *S. carpocapsae* genome in comparison with the genome of free-living *C. briggsae*, leaving open the possibility that this increased effect of balancing selection is due to Red Queen or trench warfare co-evolutionary host-pathogen dynamics. Until now, the examples of genes showing signatures of either of these evolutionary genetic interactions were limited to single genes, mostly related to effectors or genes related to the immune response. Here we found examples in which more conserved genes and genes related to mitochondrial function could also be the target of selection due to host-pathogen dynamics, opening new avenues of research. Finally, differentially expressed genes responding to host tissues, are slightly enriched in balancing selection, in agreement with the idea that this type of selection might be more relevant to genes participating in the host-pathogen interaction than other types of selection.

## Methods

### Interspecific analysis

Nucleic and amino acid sequences of eight nematode species belonging to clades IV and V [3], with parasitic and free-living lifestyles were analysed. Sequences from seven of these nematodes were downloaded from Wormbase [19] (release WS244). Sequences from *Steinernema carpocapsae* strain Breton were generated previously by our group [12] (GenBank Bioproject ID# 39853). Nematodes from clade IV included *Panagrellus redivivus* (free-living), *Strongyloides ratti* (vertebrate parasite), *Steinernema carpocapsae* (entomopathogenic), and *Bursaphelenchus xylophilus* (mycophagous) that causes the pine wilt disease [27]. Nematodes from clade V included *Caenorhabditis briggsae* and *Panagrellus redivivus* (free-living), *Heterorhabditis bacteriophora* (entomopathogenic), and *Haemonchus contortus* (vertebrate parasite).

Massive scans of positive selection were conducted in each clade, as previously described [12]. In brief, one-to-one orthologue gene sets were identified with OrthoMCL v. 2.0.7 [44]. Amino acid sequences were aligned with Clustalw2 v. 2.1 [45, 46] and concatenated. Phylogenetically informative blocks were recovered with Gblocks [47]. The best-fit evolutionary model was estimated with ProtTest [48]. Consensus phylogenetic trees were reconstructed with PhyML v. 3.0 [49]. For the analyses of positive selection, complete nucleotide sequences from each gene were aligned with RevTrans v. 1.4 [50], preserving codon homology. Signatures of positive selection were identified with Codeml from the PAML package v. 4.6 [26]. Codeml calculates ω, the ratio of nonsynonymous mutations per nonsynonymous site to the number of synonymous mutations per nonsynonymous site (ω = dN/dS) [21, 22]. Where ω >1 is indicative of positive selection, ω = 1 corresponds to the neutral expectation and ω < 1 indicates negative or purifying selection. Two models, branch and branch-site, were used to identify genes and sites under directional positive selection, using Likelihood Ratio Tests (LRTs) to assess significance. We used a script that helped with the automatization of Codeml. LRTs parameters used for each model are shown in Table 4. Significance of differences in the proportion of genes with sites under positive selection between parasitic and free-living nematodes was assessed with a *χ* ^2^ test in a 2 × 2 table.

**Table 4:** Likelihood Ratio Tests (LRTs) performed for each selected branch

Codon substitution model	LRT	Models	Parameters
Branch	H_0_: Same ω for all branches H_1_: Different ω for all branches	Model 0: one-ratio model vs Model 1: free-ratios model.	Model 0: model = 0, fix_omega = 0, omega = 0 Model 1: model = 1, fix_omega = 0, omega = 0
Branch	H_0_: Same ω for all branches H_1_: A different ω for the foreground branch	Model 0: one-ratio model vs Model 2: different ratio in the specified branch	Model 0: model = 0, fix_omega = 0, omega = 0 Model 2: model = 2, fix_omega = 0, omega = 0
Branch	H_0_: ω = 1 for the foreground branch H_1_: ω ≠ 1 for the foreground branch	Model 2: different ratio in specified branch vs Model 2 fix ω: ratio = 1 in the specified branch	Model 2: model = 2, fix_omega = 0, omega = 0 Model 2 fix ω: model = 2, fix_omega = 1, omega = 1
Branch-Site	H_0_: Same ω for all sites among branches. H_1_: Different ω for all sites in the foreground branch.	A: different ratio per site in specified branch vs A1: ω ratio = 1 per site in the specified branch	A: model = 2, NSsites = 2, fix_omega = 0 A1: model = 2, NSsites = 2, fix_omega = 1, omega = 1

NSsites = 0 for all the branch models. kappa was estimated for each gene and fixed with fix_kappa = 1 and kappa = estimated value. For all the models these parameters were the same: noisy = 3, verbose = 0,runmode = 0, seqtype = 1, CodonFreq = 2, clock = 0, aaDist = 0, icode = 0, fix_alpha = 1, alpha = 0, Malpha = 0, ncatG = 10, getSE = 0, RateAncestor = 0, Small_Diff = .5e-6, cleandata = 1, method = 1

### Intraspecific analysis

Total DNA from four different strains of *S. carpocapsae* was extracted from a pool of nematodes using phenol/chloroform extraction protocol described in [51]. DNA yield and integrity was measured with a 2100 Bioanalyzer (Agilent) using an Expert High Sensitivity DNA chip and sequenced with the Illumina HiSeq 2500 platform, at Cinvestav-Langebio Core Facilities. Details of each strain are shown in Table 5. Reads were aligned to the reference sequence [12] (GenBank Bioproject ID# 39853) with the Burrows-Wheeler Aligner (BWA) v.0.7.12 MEM algorithm [52, 53].

**Table 5:** *Steinernema carpocapsae* and *Caenorhabditis briggsae* strains used for intraspecific analysis

Species	Strain	Geographic origin	Sequencing platform	Source	Reference
*S. carpocapsae*	Breton^a^	France	454flx, SOLiD	NS	[12]
*S. carpocapsae*	All/USA strain	USA	HiSeq 2500	HGB	This study
*S. carpocapsae*	Az20	Açores, Portugal	HiSeq 2500	NS	This study
*S. carpocapsae*	Az154	Açores, Portugal	HiSeq 2500	NS	This study
*S. carpocapsae*	Az157	Açores, Portugal	HiSeq 2500	NS	This study
*C. briggsae*	AF16^a^	Guajarat, India	Combined	Wormbase	[49]
*C. briggsae*	JU1348	Kerala, India	HiSeq 2000	NCBI SRR1793004	[29]
*C. briggsae*	QR25	Quebec, Canada	GA IIx	NCBI SRR1793006	[29]
*C. briggsae*	VX0034	Hubei, China	HiSeq 2000	NCBI SRR1793007	[29]
*C. briggsae*	ED3101	Nairobi, Kenya	GA IIx	NCBI SRR1793002	[29]

Strains are natural isolates. Sequencing platform are: Combined, whole-genome shotgun sequencing (WGS) with a high-resolution, sequence-ready physical map; GA, Genome Analyzer. Libraries were Paired End (100bpx2). Strains sources are: NS, Nelson Simões; HGB, Heidi Goodrich-Blair. NCBI, National Centre for Biotechnology Information

^a^Reference genome; *S. carpocapsae* from [12], and *C. briggsae* from wormbase.org (c_briggsae. PRJNA10731.WS253) [19]

We selected *Caenorhabditis briggsae* as a model of free-living nematode for comparison because it was the only free-living species with several available genomes from different strains. Sequences from *C. briggsae* were downloaded from the NCBI Sequence Read Archive (SRA; http://www.ncbi.nlm.nih.gov/sra) and aligned with BWA-MEM to the reference genome assembly PRJNA10731 (release WS253) of *C. briggsae* strain AF16 [54], downloaded from Wormbase [19]. Accession numbers of all the strains are shown in Table 5.

SNP calling was completed with a Samtools v.1.3.1 and Bcftools v. 1.2 [55–57] pipeline. The output files, in the Variant Call Format (VCF), were used to construct haplotype map-like files [58], for each of the nematode species. These resulted from the alignment of the VCF files to the reference genomes used for the alignments. Regions with repetitive elements or with a lack of coverage were filtered.

The program Massive Tajima developed in our group (available upon request), was used to estimate the index of nucleotide diversity (π) [59], the proportional estimator of segregating sites theta (θ) [60], and Tajima’s D [30] in non-overlapping sliding windows. This program is capable of generating and analysing the observed distribution of D values. The analysis was performed across the complete genomes in windows of 1,000 bp. In windows without variation (π =0) the estimation of Tajima’s D is not possible. Windows with less than 90% of sequence coverage for one or more species were discarded. We obtained the average and the observed distribution of D values for each species. Invariable windows were excluded from the distribution. This statistic is sensitive to demographic processes that might produce signals that can be confounded with signatures of selection [13]. To ameliorate the negative demographic effects (see Discussion), we used the observed distribution to calculate corrected confidence limits with the method that Schmidt and Pool used to correct the confidence limits in simulated distributions [43]. A determined percentile on each tail of the distribution is used to find the confidence limits to identify windows with significant D values. To achieve a confidence of 95%, we intended to record the 2.5 percentile on each tail of the observed distribution. However, the D values were non-continuous (i.e., were distributed in discrete intervals, see Fig. 3), forcing us to select the 2 percentile on each tail (or jump to the 3 percentile). Thus the 2% more positive values, and the 2% more negative values were considered as candidate regions to have been targeted by selection, and established the corrected confidence limits of the distribution. For comparison, we used the theoretical distribution proposed by Tajima [30] to define the theoretical confidence limits. Significance of the difference in proportion of invariable windows between *S. carpocapsae* and *C. briggsae* was assessed with a *χ* ^2^ test in a 2 × 2 table. The differences in theta and Tajima’s D averages were assessed by *t*-test and a Wilcoxon (W) test. The difference in the distribution of D values was assessed by the nonparametric Kolmogorov-Smirnov (K-S) test.

### Selection in differentially expressed proteins

Shotgun proteomics data from nematodes induced with insect tissues and from controls without induction were generated previously [12]. In brief, IJ nematodes were induced with either haemolymph or intestine of *Galleria mellonella* for 4 hours. Nematodes were ground in liquid nitrogen and used to extract total soluble proteins. Shotgun proteomics was done fractioning 200 μl of the total protein extract. Thirty μg of protein from each fraction were then analysed by LC-MS/MS using a Thermo Scientific Q-Exactive Orbitrap MS spectrometer in conjunction with a Proxeon Easy-nLC II HPLC (Thermo Scientific) and a source Proxeon nanospray using a reverse phase column. The MS/MS spectra were acquired using the TOP15 method following the equipment manufacturer’s instructions. All analyses were run in duplicate, including treatment samples and controls [12]. With this data, Rougon-Cardoso et al. [12] conducted a qualitative analysis in which those proteins that were expressed in the samples and absent (below the detection level) in the controls, or vice versa, were detected. In the present study, using the same data, we conducted a label-free quantitative analysis, in which those proteins expressed only in the samples or the controls were excluded. Raw files of every fraction of the samples were processed using MaxQuant v 1.5.2.8 [61, 62] for protein identification and quantification. For identification of proteins, a false discovery rate of 1% at the peptide and protein level was used. The average absolute mass deviation was 0.2 parts per million (p.p.m.). For protein quantification, we used intensity based absolute quantification, or iBAQ [63]. Proteins amounts were calculated as the sum of all peptide peak intensities divided by the number of theoretically observable tryptic peptides. Data analysis was performed with Microsoft Office Excel and Perseus v.1.5.1.6 [64]. Differential expression analysis was performed using only proteins observed in the two replicates per condition, using *t*-test analyses and a False Discovery Rate (FDR) of 5%. The results of the qualitative analysis done in [12] were combined with the results of the quantitative label-free analysis described above.

The total list of differentially expressed proteins were contrasted against the list of genes with signatures of selection from both the interspecific and the intraspecific analyses, to identify the number of genes with expression data having also evolutionary data. From these genes, we counted the number of genes showing specific signatures of selection in both the differentially expressed and non-differentially expressed genes. These values were compared against each other and against the global analysis (inter and intraspecific). The significance of these differences were assessed by Fisher’s exact tests in 2 × 2 tables.

### Functional annotation and enrichment analyses

The association of genes to gene ontology (GO) terms (functional annotation) and enrichment of GO categories in genes with signatures of selection were performed with Blast2GO with a Fisher’s exact test [65]. For the enrichment tests, we assessed if in genes with signatures of selection there were overrepresented GO terms in comparison to the genes without signs of selection in each analysis. In this way, we compared the associated GO terms found among genes with signatures of positive selection against the associated GO terms found in the rest of the analysed orthologue genes. In addition, GO terms in genes associated with windows with significant Tajima’s D values were contrasted with the remaining genes in the genome.

### Genetic Distances

Genome sequences were aligned gene by gene and concatenated for *S. carpocapsae* and *C. briggsae* strains. Nucleotide​ ​substitution​ ​models​ ​​were estimated with jModelTest​ v. 2.1.3​ [66], and the best model under the Bayesian​ ​information​ ​criterion​ ​(BIC) was chosen (Additional file 12). Alignments were transformed into phylip format with Clustalw2 v. 2.1 [45, 46], and pairwise​ ​maximum​ ​likelihood​ ​distances were estimated between strains with Tree-puzzle​ [67].

## Additional files

Additional file 1: Functional annotation of *S. carpocapsae* genes with sites evolving under positive selection. Donut chart showing the GO term distribution at level 4 for biological process (BP), molecular function (MF), and cellular component (CC). GO analysis was performed using Blast2GO [65]. (PDF 458 kb)

Additional file 2: GO Distribution by Level 4 of *S. carpocapsae* genes with sites evolving under positive selection. (XLSX 20 kb)

Additional file 3: Enriched GO terms in the genes with sites under positive selection in nematode clade IV. (XLSX 13 kb)

Additional file 4: Enriched GO terms in the genes with sites under positive selection in nematode clade V. (XLSX 14 kb)

Additional file 5: Over-represented unique GO terms in genes with sites under positive selection. a-b, enriched GO terms by lifestyle and d-h by species. a, parasites from clade IV; b parasites from clade V; c, free living in clade IV; d *Steinernema carpocapsae*; e, *Strongyloides ratti*; f, *Panagrellus redivivus*; g, *Heterorhabditis bacteriophora*; h, *Haemonchus contortus*. Biological process in green, molecular function in blue, and cellular component in yellow. Enrichment analyses were performed with Blast2GO with a Fisher’s exact test [65]. (PDF 213 kb)

Additional file 6: *Steinernema carpocapsae* protein coding genes included in windows with significant Tajima’s D values. (XLSX 71 kb)

Additional file 7: Functional annotation of *S. carpocapsae* genes in windows with significant Tajima’s D value. Donut chart showing the GO term distribution at level 4 for biological process (BP), molecular function (MF), and cellular component (CC) of the genes in windows with significant Tajima’s D value. GO analysis was performed using Blast2GO [65]. (PDF 482 kb)

Additional file 8: GO Distribution by Level 4 of *Steinernema carpocapsae* protein coding genes included in windows with significant Tajima’s D values. (XLSX 33 kb)

Additional file 9: Differentially expressed proteins in *S. carpocapsae* infective juveniles induced with insect tissues. (XLSX 131 kb)

Additional file 10: Non-differentially expressed proteins in *S. carpocapsae* infective juveniles induced with insect tissues. (XLSX 232 kb)

Additional file 11: Genes for differentially expressed proteins with sites under positive selection or significant Tajima’s D values. (XLSX 85 kb)

Additional file 12: Matrices of Maximum Likelihood distances between the analysed strains of *S. carpocapsae* and *C. briggsae*. (XLSX 12 kb)
