# Supplementary material for: Signatures of co-evolutionary host-pathogen interactions in the genome of the entomopathogenic nematode Steinernema carpocapsae
Source: BMC Evol Biol. 2017 Apr 26;17:108. doi: 10.1186/s12862-017-0935-x (PMC5405473; doi:10.1186/s12862-017-0935-x)

Additional File 1

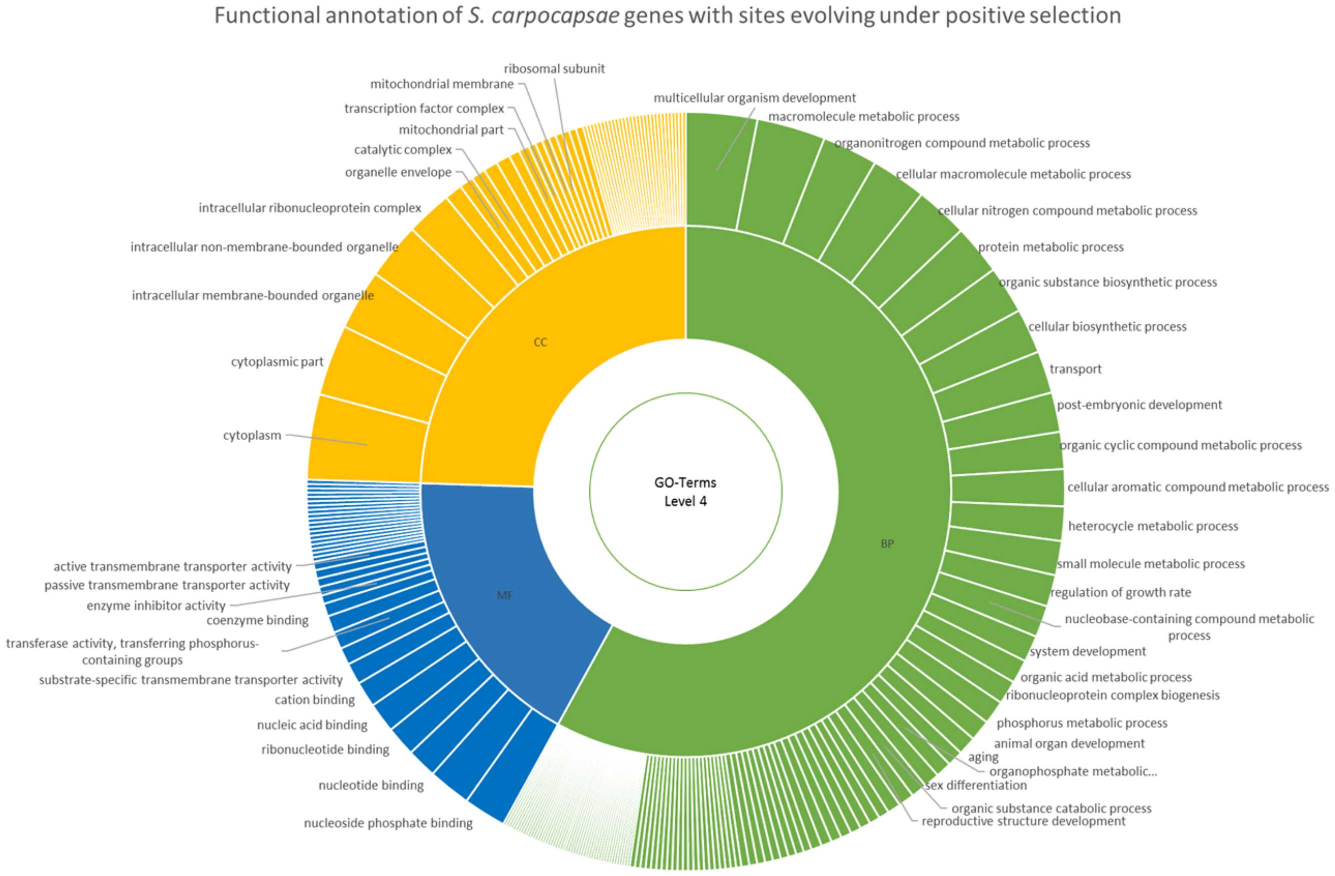

Supplement: Supplementary file 1 — Functional annotation of S. carpocapsae genes with sites evolving under positive selection. Donut chart showing the GO term distribution at level 4 for biological process (BP), molecular function (MF), and cellular component (CC). GO analysis was performed using Blast2GO [65]. (PDF 458 kb) [file 12862_2017_935_MOESM1_ESM.pdf]
