# Supplementary figures and images for: Signatures of co-evolutionary host-pathogen interactions in the genome of the entomopathogenic nematode Steinernema carpocapsae
Source: BMC Evol Biol. 2017 Apr 26;17:108. doi: 10.1186/s12862-017-0935-x (PMC5405473; doi:10.1186/s12862-017-0935-x)

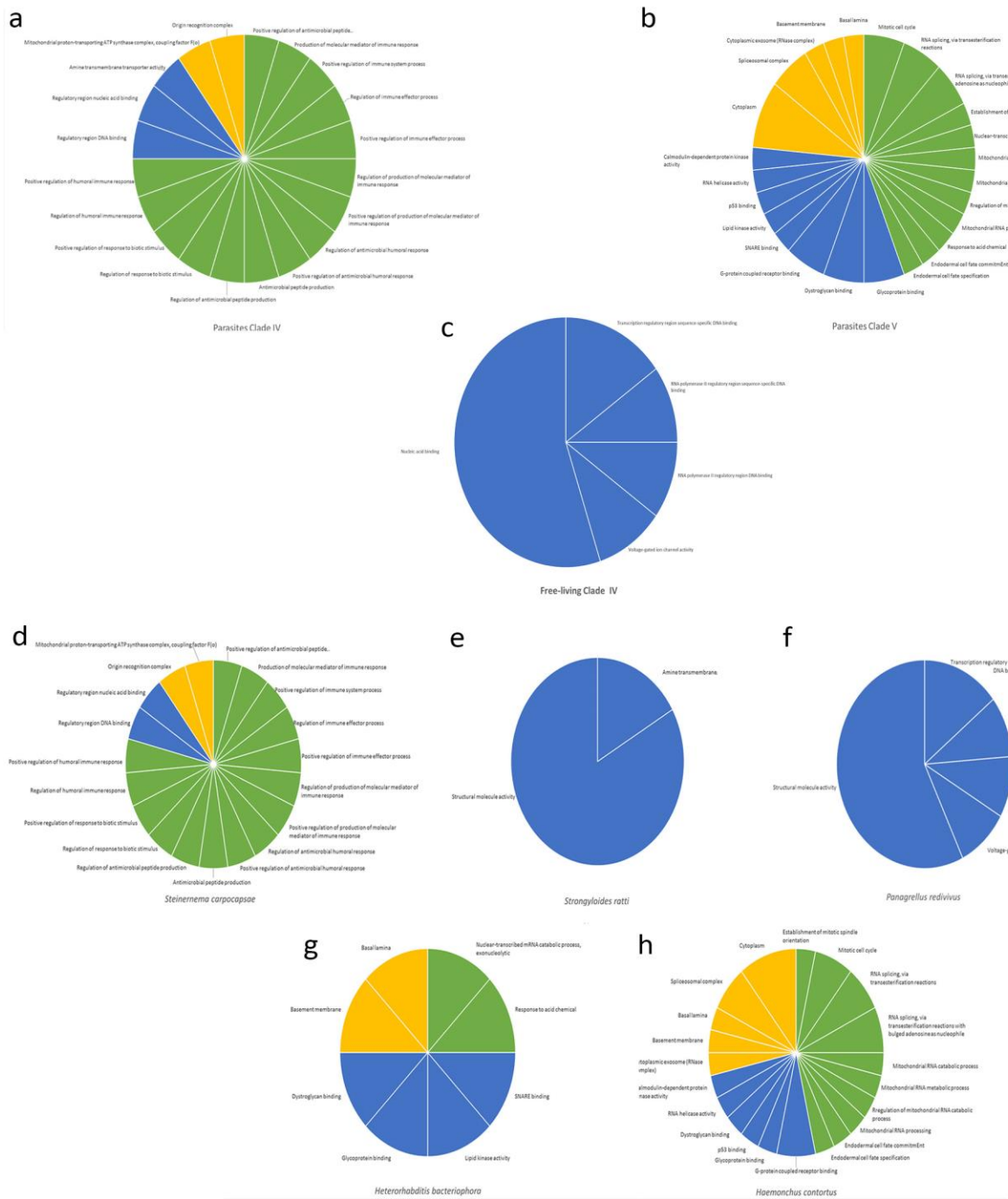

Supplement: Supplementary file 5 — Over-represented unique GO terms in genes with sites under positive selection. a-b, enriched GO terms by lifestyle and d-h by species. a, parasites from clade IV; b parasites from clade V; c, free living in clade IV; d Steinernema carpocapsae; e, Strongyloides ratti; f, Panagrellus redivivus; g, Heterorhabditis bacteriophora; h, Haemonchus contortus. Biological process in green, molecular function in blue, and cellular component in yellow. Enrichment analyses were performed with Blast2GO with a Fisher’s exact test [65]. (PDF 213 kb) [file 12862_2017_935_MOESM5_ESM.pdf]
